# PLGA-PEG Nanoparticles Show Minimal Risks of Interference with Platelet Function of Human Platelet-Rich Plasma

**DOI:** 10.3390/ijms21249716

**Published:** 2020-12-19

**Authors:** Rana Bakhaidar, Sarah O’Neill, Zebunnissa Ramtoola

**Affiliations:** School of Pharmacy and Biomolecular Sciences, RCSI, University of Medicine and Health Sciences, D02 YN77 Dublin 2, Ireland; ranabakhaidar@rcsi.ie (R.B.); soneill@rcsi.com (S.O.)

**Keywords:** PLGA-PEG, nanoparticles, particle size, platelet rich plasma, platelet activation, platelet aggregation

## Abstract

The expansion of nanotechnology for drug delivery applications has raised questions regarding the safety of nanoparticles (NPs) due to their potential for interacting at molecular and cellular levels. Although polymeric NPs for drug delivery are formulated using FDA-approved polymers such as lactide- and glycolide-based polymers, their interactions with blood constituents, remain to be identified. The aim of this study was to determine the impact of size-selected Poly-lactide-co-glycolide-polyethylene glycol (PLGA-PEG) NPs on platelet activity. The NPs of 113, 321, and 585 nm sizes, were formulated and their effects at concentrations of 0–2.2 mg/mL on the activation and aggregation of platelet-rich plasma (PRP) were investigated. The results showed that NPs of 113 nm did not affect adenosine diphosphate (ADP)-induced platelet aggregation at any NP concentration studied. The NPs of 321 and 585 nm, at concentrations ≥0.25 mg/mL, reduced ADP-activated platelet aggregation. The platelet activation profile remained unchanged in the presence of investigated NPs. Confocal microscopy revealed that NPs were attached to or internalised by platelets in both resting and activated states, with no influence on platelet reactivity. The results indicate minimal risks of interference with platelet function for PLGA-PEG NPs and that these NPs can be explored as nanocarriers for targeted drug delivery to platelets.

## 1. Introduction

The past decade has witnessed a heightened interest in nanoparticles (NPs) as drug delivery systems (DDS) for the controlled and site-specific release of drugs. Essentially, NPs have provided an opportunity to explore new avenues in diagnosis, cellular and molecular imaging, prevention, and therapy of various diseases. In addition, drug-loaded NPs can, then, be delivered effectively to the desired target site without compromising the activity of incorporated therapeutics [1,2,3,4]. Recently, studies have shown the possibilities of nano-based DDS for administration through vascular pathways, such as the long-circulating Poly-lactide-co-glycolide-polyethylene glycol (PLGA-PEG)-based NPs. When such nanocarriers enter the vasculature, blood components are more likely to be exposed to the bare NPs’ surfaces; this enhances their potential for interacting at both a molecular and cellular level for longer time periods [5]. Hence, it is of prime significance to identify the impact of the physicochemical characteristics of these nano-entities, such as particle size (PS) and zeta potential (ZP), on blood components and, in particular, on platelets. It is also important to understand how any modification of NP properties may enhance their efficacy, as well as their biological and toxicological profiles [2]. Several authors have pointed out the pro-aggregatory/activation effects of some NPs on platelets, while other studies have presented their antiplatelet effects. This consequently raises questions regarding the safety of nanomaterials, particularly, in terms of interference with haemostatic equilibrium [6,7]. The interaction of NPs with platelets may lead to disruption of normal platelet function, predominantly resulting in the inadvertent generation of a thrombus leading to a cardiovascular event, such as myocardial infarct or stroke [8]. We previously reported the dependency of size and concentration of PLGA-PEG NPs on the aggregation profile of washed platelets (WPs) and showed a delay in the aggregation profile of WPs in the presence of NPs of 348 and 576 nm at NP concentrations of 1.5–2.2 mg/mL [9].

In this study, our focus was to examine the potential of PLGA-PEG NPs, in similar size ranges used in our previous study, to impact on platelet function in platelet-rich plasma (PRP). PRP is a more physiologically relevant milieu as compared with the WPs system in which plasma proteins are absent. In addition to plasma proteins, such as fibrinogen, PRP contains a number of growth factors including platelet-derived growth factors, platelet-derived angiogenesis factor (PDAF), vascular endothelial growth factor, and may also contain leukocytes synthesising interleukin-1 (IL-1) which aids in immune response [10,11]. As compared with WPs, the response of platelets in PRP in the presence of NPs might vary considerably, as these additional components are present and would have been absent when WPs were studied previously [8]. 

Therefore, we examined the effect of size and concentration of the PLGA-PEG NPs on the two interlinked platelet functions of platelet aggregation and activation. Adenosine diphosphate (ADP) was selected to induce platelet aggregation and activation, in our studies, as it is naturally present in the human body and plays a role in maintaining normal haemostasis and thrombosis. Defects in the binding of ADP to its receptor, which may be a result of interaction with a foreign biomaterial introduced into the human body, warrants attention as it may lead to pathophysiological impacts [12,13]. In addition, we investigated the interaction of the size-selected NPs with platelets in PRP in both resting and active states, using confocal laser scanning microscopy (CLSM).

## 2. Results

### 2.1. Effect of Size and Concentration of Poly-Lactide-Co-GlycolidePpolyethylene Glycol (PLGA-PEG) Nanoparticles (NPs) on Aggregation of Platelets in Platelet-Rich Plasma (PRP)

Using the formulation parameters as those used in our previous study, PLGA-PEG NPs of three different sizes similar to those previously reported, were obtained [9]. The average particle size (PS) and polydispersity index (PDI) obtained were 113 ± 7.22, 321 ± 46.43, and 585 ± 5.87 nm and 0.10 ± 0.03, 0.55 ± 0.07, and 0.67 ± 0.03 respectively, for PLGA-PEG concentrations of 10, 55, and 100 mg/mL. Statistical analysis confirmed a highly significant difference between the particle sizes of the three batches, ensuring a reliable size-related comparison for examining their effects on platelets in PRP (*p* = 0.0016 and 0.0006 comparing PS of batch 1 and 2 with batches 2 and 3, respectively).

Data from platelet aggregation assays showed that during the 4 min incubation time of NPs with unstimulated platelets in PRP, no spontaneous platelet aggregation was recorded for any of the NP sizes or concentrations (0.01–2.2 mg/mL) tested as compared with the control (0 ± 0% PA). In the absence of NPs, platelets in PRP incubated with ADP at 20 µM, resulted in % PA ranging between 85.25 ± 1.18% and 87.64 ± 10.24% (*n* = 4–5) (Table 1). Despite interindividual variability in response to ADP, the % PA observed in this study was similar to those values reported to be within the normal range documented in several previous studies of large cohorts of normal volunteers [14].

**Figure 1:**
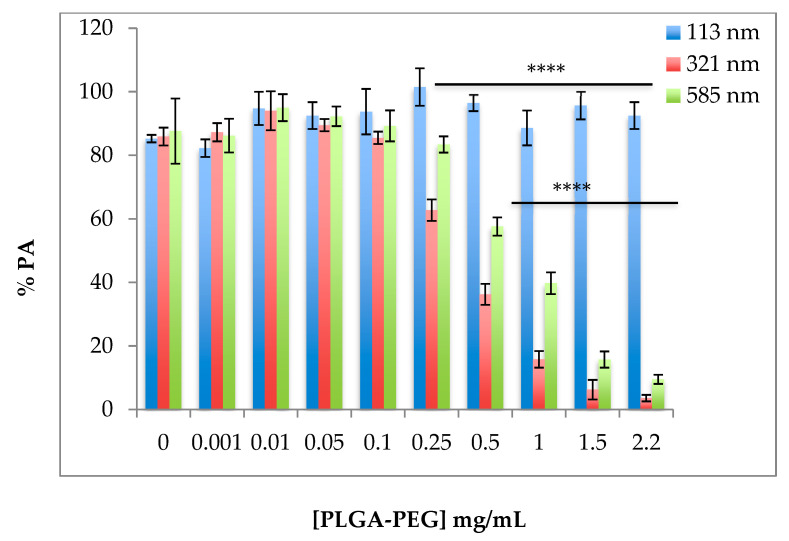
Effect of size of PLGA-PEG NPs on percent platelet aggregation (% PA) of platelets in PRP induced by ADP; ADP was added at a final concentration of 20 µM for 12 min after incubation with PLGA-PEG NPs of different sizes for 4 min. Values are mean of *n* = 4–5 ± SEM. One-way ANOVA and Tukey’s multiple comparison tests were used for analysis. **** *p* < 0.0001 for 321 and 585 nm NPs at ≥0.25 and ≥0.50 mg/mL respectively.

Following the addition of ADP, on the one hand, no significant change in % PA was observed in PRP incubated with NPs of 113 nm at all concentrations tested (Table 1, Figure 1, *p* > 0.05, mean of *n* = 4–5 ± SEM). On the other hand, a significant decrease in % PA was noted in the presence of 321 nm NPs at concentrations of ≥0.25 mg/mL (Table 1, Figure 1, *p* < 0.0001). This inhibitory effect increased with increasing NP concentration, with the greatest inhibitory effect observed at 2.2 mg/mL (% PA = 3.6 ± 1.03). Similarly, NPs of 585 nm considerably reduced % PA in PRP induced by ADP at NP concentrations of 0.5 to 2.2 mg/mL (Table 1, Figure 1).

### 2.2. Effect of the Size of PLGA-PEG NPs on the Activation of Platelets in Platelet-Rich Plasma (PRP)

**Figure 2:**
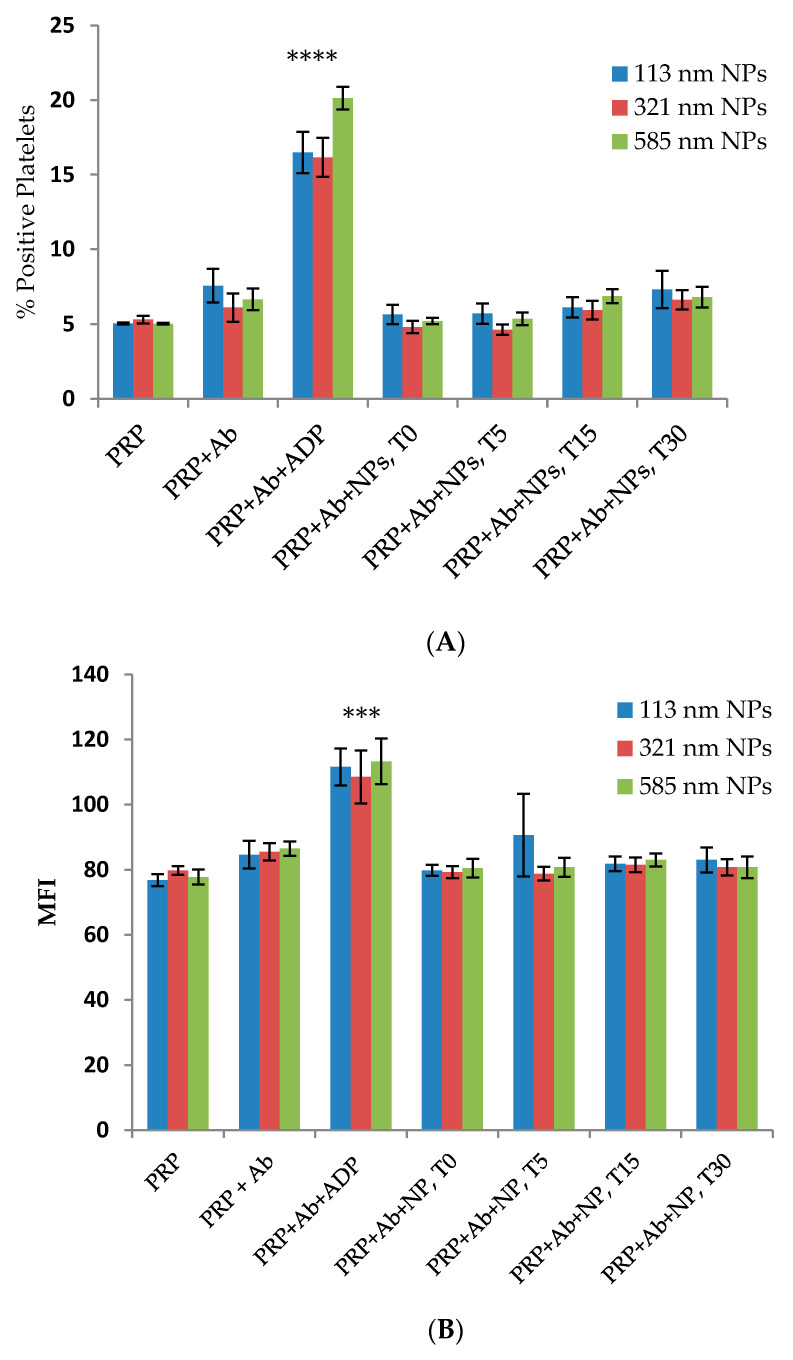
Flow cytometry analysis of the effect of size of PLGA-PEG NPs at 2.2 mg/mL on platelet activation over time (T) in minutes. (**A**) % Positive platelets (% PP); (**B**) Mean fluorescence intensity (MFI). NPs were incubated with aliquots of resting platelets in PRP for 4 min without stirring prior to the addition of anti-CD62P antibody (10 µg/mL). Then, the reaction was terminated using 1% *v*/*v* FA at the specified time (T) intervals. % Positive platelets represents the percentage CD62P-positive platelets in the total platelet population. *** *p* < 0.001 **** *p* < 0.0001 as compared with PRP+Ab (control) one-way ANOVA followed by post hoc Tukey test.

A significant increase in the expression of CD62P, on the platelet surface of ADP-activated platelets incubated with anti-CD62P antibody, was observed (% PP = 21.77 ± 4.64%, *p* < 0.005) as compared with resting platelets in PRP incubated with anti-CD62P antibody. In the presence of the different sized NPs, the flow cytometry analysis showed that over the incubation time of 30 min, none of the different-sized PLGA-PEG NPs exerted a stimulatory or an inhibitory effect on the expression of CD62P on the surface of resting platelets in PRP as compared with the controls (*p* > 0.05, *n* = 4–6, Figure 2A,B).

**Figure 3:**
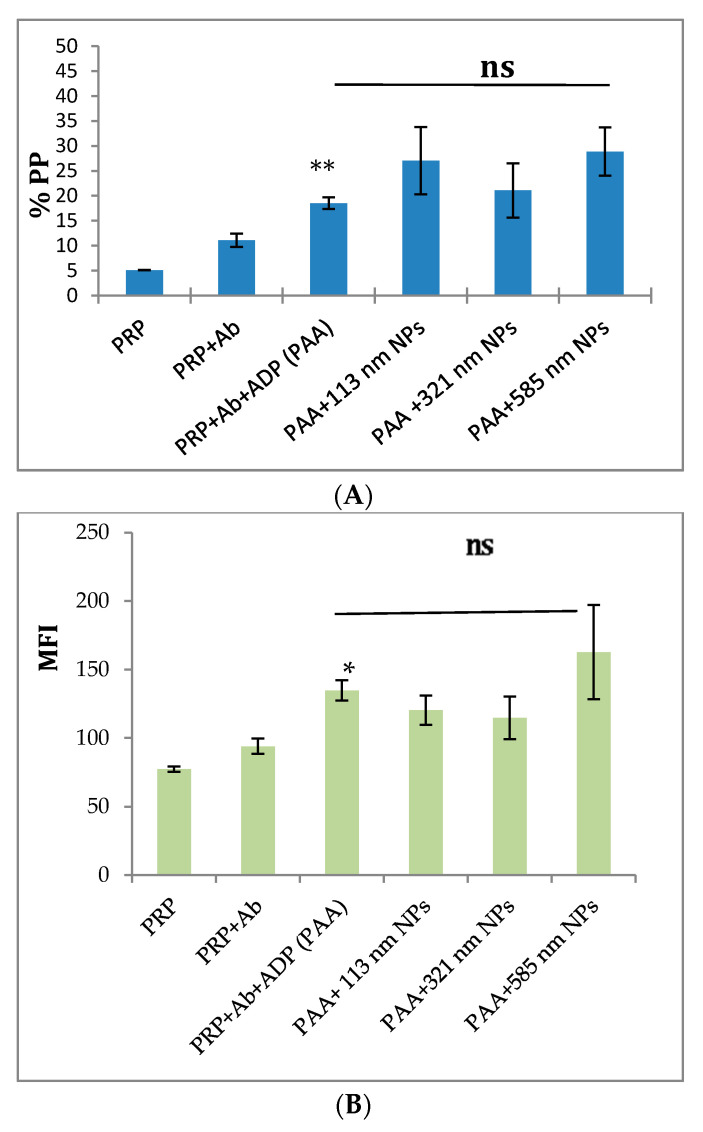
Flow cytometry analysis of the effect of size on PLGA-PEG NPs, at 2.2 mg/mL, on ADP activation of PRP. (**A**) % Positive platelets (% PP); (**B**) Mean fluorescence intensity (MFI). Data are the average of *n* = 3–5 ± SEM. The NPs were pre-incubated with aliquots of platelets in PRP, followed by activation using 20 µM ADP. Samples were, then, incubated in the presence of CD62P antibody (10 µg/mL). % Gated platelets represent the percentage of CD62P-positive platelets in the total platelet population. * *p* < 0.05, ** *p* < 0.01 as compared with PRP+Ab+ADP (control), one-way ANOVA followed by post hoc Tukey test, ns = not significant.

ADP strongly increased the expression of CD62P on the platelet surface (*p* < 0.05, mean of *n* = 3 ± SEM). The incubation of 113, 321, and 585 nm NPs with platelets in PRP did not result in any significant elevation or reduction in the expression of CD62P on the platelet surface following addition of ADP (Figure 3A,B).

### 2.3. Confocal Imaging of the Interaction of PLGA-PEG NPs with Platelets in PRP

**Figure 4:**
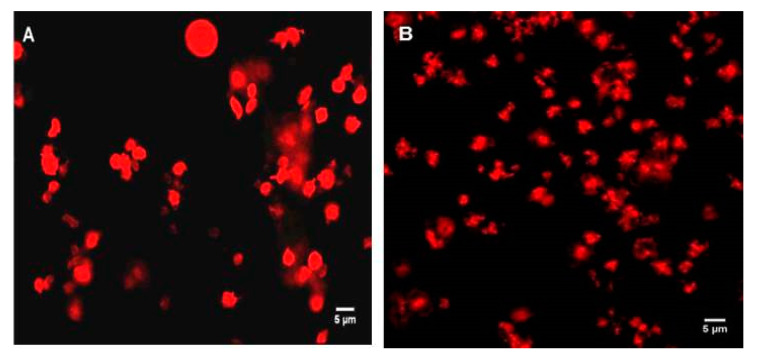
Representative confocal microscopy images. (**A**) Resting platelets in PRP; (**B**) ADP-induced platelets in PRP. Platelets in PRP were fluorescently labelled with phalloidin-TRITC at 0.25 mg/mL and fixed using 1% formaldehyde. Magnification 100×. Scale bar represents 5 µm.

Coumarin-labelled PLGA-PEG NPs of 113, 321, and 585 nm, at a final NP concentration of 2.2 mg/mL, were incubated with unactivated platelets in PRP at increasing time intervals of 1, 5, and 30 min. Platelets in PRP appeared as classical “resting” platelets, displaying a uniform discoid shape (Figure 4A) and, in the presence of ADP, showed the characteristic typical shape of activated platelets with extended filopodial projections (Figure 4B).

**Figure 5:**
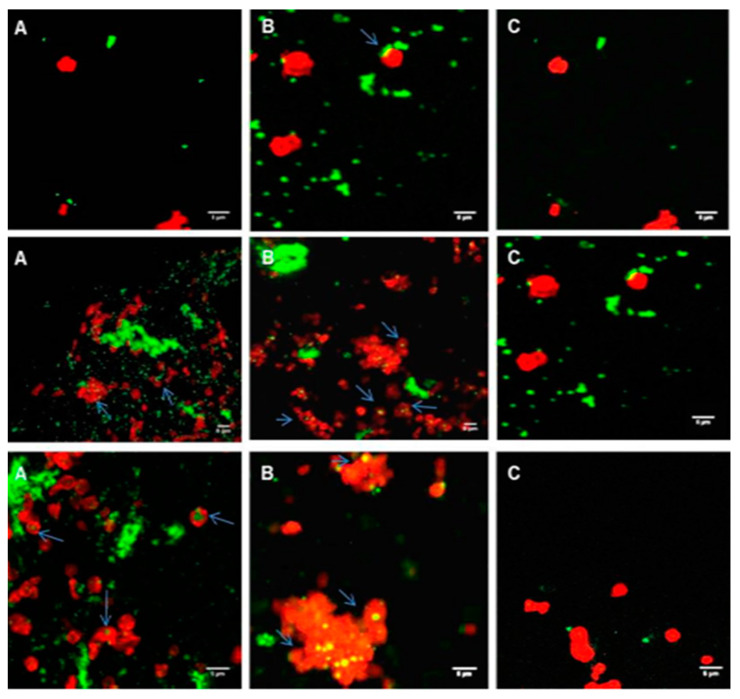
Confocal microscopy images of platelets in PRP (red) incubated with coumarin-labelled PLGA-PEG NPs (green) at 2.2 mg/mL. (**A**) For 1 min; (**B**) For 5 min; (**C**) For 30 min. Platelets in PRP were fluorescently labelled with phalloidin-TRITC at 0.25 mg/mL and fixed using 1% formaldehyde Upper panel, 113 nm NPs; middle panel, 321 nm NPs; lower panel, 585 nm NPs. Magnification 100×, scale bar represents 5 µm.

Incubation of PLGA-PEG NPs with “resting” platelets in PRP did not result in any apparent platelet activation, as the unactivated (resting) shape of platelets was maintained over the incubation time tested (Figure 5A–C), despite the close proximity and association of platelets and NPs and possible internalisation of NPs by the platelets (Figure 5A–C).

**Figure 6:**
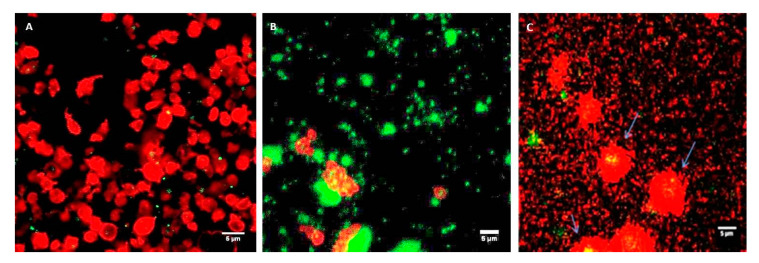
Confocal microscopy images showing ADP-stimulated platelets in PRP (red) incubated with coumarin-labelled PLGA-PEG NPs (green). (**A**) 113 nm; (**B**) 321 nm; (**C**) 585 nm, at 2.2 mg/mL NPs. Platelets in PRP were fluorescently labelled with phalloidin-TRITC at 0.25 mg/mL and fixed using 1% formaldehyde. Magnification ×100, scale bar represents 5 µm.

Incubation of PLGA-PEG NPs, with platelets in PRP stimulated by ADP, did not appear to influence the activation of the platelets. The characteristic activated platelet morphology similar to the control activated platelets in PRP (Figure 4B) was observed, irrespective of the size of NPs (Figure 6A–C). Confocal images in Figure 6A–C showed close proximity and association of NPs with platelets, and possible internalisation of the NPs by the platelets.

## 3. Discussion

The interaction between the size-selected NPs and platelets in PRP showed that prior to the addition of the agonist, ADP, pre-incubation of platelets in PRP with increasing concentration of different-sized PLGA-PEG NPs did not result in any spontaneous platelet aggregation. This was in agreement with studies undertaken by Ramtoola et al. [2] where non-pegylated PLGA NPs were reported to not influence aggregation of unstimulated platelets in PRP at concentrations ranging from 100 to 500 µg/mL [2]. Carboxylate-modified polystyrene NPs at variable concentrations were also reported to not modify resting platelet aggregation profile [15]. Positively charged 2.8 nm and negatively charged 4.8 nm quantum dots (QDs), which are used for biologically oriented applications including biomedical imaging, drug delivery, and photodynamic therapy, were also reported to not cause significant activation of platelets in PRP [16,17].

Following the addition of the agonist, ADP, to platelets incubated with 113 nm PLGA-PEG NPs, no significant effect on % PA was observed. This was in agreement with published data as a recent study suggested that exposure of PRP to colloidal gold NPs of 30 and 50 nm hydrodynamic sizes did not show any effect on untreated or collagen-treated platelets in PRP [18]. Another published study investigating gold NPs of small sizes (13, 20, and 29 nm) indicated no effect on platelet aggregation in PRP induced by 15 µM ADP [19]. Interestingly, nano-silver particles of 13–15, 30–35, and 40–45 nm were reported to exhibit an inhibitory effect on platelet aggregation in PRP induced by ADP at 15 µM, all to a similar extent [20].

In our study, NPs of 321 and 585 nm at concentrations of 0.25 mg/mL and above reduced % PA of platelets in PRP, induced by ADP. This may be as a result of their physical presence in the medium, forming a physical barrier between platelets, thereby reducing the normal platelet aggregation response to the agonist ADP. NPs of 321 nm showed a higher reduction in % PA of platelets in PRP. This was related to the greater tendency of the 321 nm NPs to aggregate as compared with the larger-sized NPs of 585 nm [9]. This high heterodispersity of the 321 nm NPs may have resulted in the formation of a larger physical barrier to the passage of light through the sample during measurement, hence, giving a lower % PA for these NPs. The results observed in this study were similar to the platelet aggregometry data for thrombin-activated WPs incubated with various sized NPs which we reported previously [9]. The response of platelets in PRP in the presence of NPs did not appear to vary despite the additional components present in PRP.

PS has been reported to play a pivotal role in inducing or reducing platelet activation [21]. Particles of different sizes are capable of binding to different plasma proteins which could lead to different levels of platelet activation [22]. A number of factors appear to contribute to the capacity for NPs to influence platelet activation and aggregation [19]. Other studies have similarly suggested a weak inhibition of platelets in PRP aggregation induced by collagen by 21.3 ± 3.3% and by the synthetic peptide thrombin receptor activating peptide (TRAP) by 23.1 ± 3.2%, as a result of the presence of PLGA NPs of 640 nm size at 0.1 mg/mL concentration, an observation explained by either reduced platelet-platelet interaction or agonist binding [8]. An alternative explanation to the observed inhibition of platelet aggregation may possibly be the outcome of specific modulation of platelet signalling cascade, or an effect on the binding of fibrinogen to its platelet receptor αI_Ib_β_3_.

Platelet activation and aggregation are intricately interlinked, since platelet activation has been reported to be essential for aggregation to take place [23]. The binding of the fluorochrome labelled antibody to CD62P on the platelet surface is a marker of platelet activation [24]. The flow cytometry data in this study showed no alteration in the expression of CD62P on the surface of either resting or ADP-induced platelets in the PRP samples incubated with PLGA-PEG NPs of the different sizes at 2.2 mg/mL concentration, in the presence of a saturating concentration of anti-CD62P antibody.

In a biological medium such as human blood or human blood fractions such as PRP, NPs encounter the complex environment of the plasma and may become coated with serum proteins forming “the protein corona”. This could change the NP surface properties, promoting their interaction with blood entities, thereby dictating the biological response to these nano-entities, resulting, for instance, in a change in platelet reactivity or NPs fate or route of clearance [25,26].

NP characteristics such as PS, PDI, hydrophobicity/hydrophilicity, shape, roughness, chemical composition, heterogeneity, surface crystallinity, and surface charge may actively contribute to the interaction with plasma proteins leading to platelet activation [21,22]. Mayer and co-workers [27] suggested that differences in PS amongst NPs had influenced the degree of platelet activation determined by the expression of the anti-CD62P antibody on their surfaces. In their study, they observed 26 nm carboxylated-polystyrene NPs tended to activate platelets at 0.5 mg/mL concentration, whereas particles of a larger size did not impact on platelet activation. The variable capacity of polystyrene NPs to bind plasma proteins in blood was noted by other researchers, resulting in different levels of platelet activation. Nanoparticles, shown to inhibit platelet activation, appeared to coat platelets and act as a barrier limiting CD42b phycoerythrin (PE) and CD62P Alexa Fluor 488-labelled antibodies access to platelet surface [22].

Confocal microscopy, used in this study to visualise the interaction between the NPs and platelets in unactivated and activated PRP, confirmed the characteristic changes of platelet morphology following the addition of ADP. Throughout the incubation period, with the various sizes of NPs, the “resting” platelet morphology remained unchanged. Other studies have similarly concluded this observation. In one study, small (87 nm) and intermediate sized (312 nm) latex NPs, as well as metal NPs, appeared to be engulfed by the platelets, while they retained their discoid shape [28]. In our study, the addition of NPs to platelets in PRP in the presence of ADP did not inhibit platelet activation, as confirmed by the characteristic activated platelet morphology observed in Figure 6A–C. The NP association with activated platelets in PRP was similar to the interaction observed with resting platelets in PRP. Confocal imaging also showed possible internalisation of NPs within the platelets, in particular, of the larger NP size of 585 nm. In our previous studies with WPs, NP association and possible internalisation into WPs was also reported [9].

In other studies, the internalisation of gold NPs (Au NPs), ranging between 20 and 70 nm by resting platelets was reported to be highly dependent on the size [29]. The immediate engulfment of NPs such as thorium dioxide; fibrinogen-coated gold; small, medium sized, large, and very large latex spherules upon their incubation with PRP; or WPs have also been reported [30]. Recently, the internalisation of pre-activated latex microspheres following a 1 h of incubation with platelets in PRP has been shown, as prior activation was recognised to permit shape change and subsequent pseudopodia formation, through which the microspheres were transported inside platelets [31]. Platelets are capable of engulfing proteins, as well as various particulates such as viruses and bacteria as part of their role in host defence in the human body, as well as a number of NPs of different physicochemical properties through different mechanisms [32].

## 4. Materials and Methods

### 4.1. Materials

Adenosine 5′-diphosphate monopotassium salt dihydrate (ADP), coumarin-6 (98%), fibrinogen from human plasma, Polysorbate 80 (Tween^®^ 80), sodium citrate (Tribasic, 10 dehydrate (HOC(COONa)(CH_2_COONa_2_), phalloidin-tetramethylrhodamine isothiocyanate (TRITC) dye, formaldehyde solution (37% *w/v* % in water) were purchased from Sigma-Aldrich Dublin, Ireland. PLGA-PEG containing PEG at 10% *w/w* (poly-d, l-lactic-co-glycolic acid-polyethylene glycol diblock copolymer 50:50 mPEG and 33 kDa with inherent viscosity 0.05–0.15 dL/g; Lakeshore Biomaterials, was purchased from Evonik Industries AG, Essen, Germany. Deionised water was used throughout the experiments. Phycoerythrin (PE)-labelled mouse anti-Human CD62P antibody (1.5 µg/mL was purchased from Beckton Dickinson (BD), Oxford, UK.

### 4.2. Preparation and Characterisation of PLGA-PEG NPs

PLGA-PEG NPs containing the fluorescent marker, coumarin-6, were formulated using the solvent dispersion technique, as previously described [9]. Briefly, PLGA-PEG polymer was dissolved in acetone to form 10, 55, and 100 mg/mL solutions. Coumarin-6 at 0.05% *w/w* of the polymer was dissolved in the PLGA-PEG solutions. The polymer solutions were added dropwise to an external aqueous phase containing the surfactant Tween 80^®^ at 2% *w/v*, under constant stirring. The NPs formed were recovered through centrifugation at 23,600× *g* for 20 min. The average PS, polydispersity index (PDI), and ZP of the NPs were determined by dynamic light scattering (DLS) technique, using a Malvern Zetasizer Nano ZS 90 (Malvern Instruments, Worcestershire, UK).

### 4.3. Preparation of Platelet-Rich Plasma (PRP)

Venous whole blood was obtained from healthy human volunteers, who had abstained from taking aspirin and other anti-platelet agents for the previous 7–10 days. Ethical approval was obtained for this study from the Research Ethics Committee of the Royal College of Surgeons in Ireland. The blood was centrifuged at 150 *g* for 12 min, at room temperature, to obtain the platelet-rich plasma (PRP), which was removed to a separate container. The platelet count of the PRP was made using a Sysmex-K1000 counter (Toa medical Electronics Company Ltd., Kobe, Japan). The remaining blood was centrifuged at 720 g for 12 min to produce platelet-poor plasma (PPP).

### 4.4. Effect of Size and Concentration of PLGA-PEG NPs on Platelet Aggregation

Platelet aggregation was determined by measuring the change in the optical density of stirred PRP in the absence or presence of 2.2 mg/mL, a concentration suitable for drug delivery purposes, PLGA-PEG NPs of variable sizes, following the addition of the platelet agonist, ADP. PRP was incubated with NPs for 4 min prior to the addition of ADP, at 20 µM final concentration and for 12 min post addition of ADP. Platelet aggregation was monitored in an eight-channel platelet aggregometer, PAP-8 (Bio/Data Corporation, Horsham, PA, USA). Aggregation results were expressed as final percent aggregation (% PA) at the end of the reaction time of 12 min. Data are presented as mean of *n* = 4–6 ± SEM. Changes in light transmission were recorded against PPP, which represented 100% light transmission.

### 4.5. Effect of Size and Concentration of PLGA-PEG NPs on Platelet Activation

PRP samples (250 × 10^3^/µL) were incubated with 20 µL phycoerythrin (PE)-labelled CD62P antibody and mixed with PLGA-PEG NPs of different sizes, in the presence of 20 µM ADP and incubated for 4 min at 37 °C. PRP was also mixed with NPs of different sizes and CD62P in the absence of ADP and incubated over 30 min at 37 °C. At predetermined time points, samples were fixed using 1% *v/v* formaldehyde (FA) and platelet activation was measured by flow cytometry. Platelet activation was determined by measuring the level and extent of CD62P antibody binding to the platelet surface, expressed as percent positive cells (% PP) and mean fluorescence intensity (MFI). Platelets with and without CD62P were used as negative controls and platelets activated with ADP at 20 µM for 4 min was the positive control. Data are average of *n* ≥ 3 ± SEM.

### 4.6. Confocal Microscopy of the Interaction of PLGA-PEG NPs with PRP over Time

Coumarin-labelled NPs, at 0.1 and 2.2 mg/mL, a range of concentrations reported earlier for drug delivery, were incubated with PRP for 1, 5, 15, and 30 min. At each time point, samples were fixed with 1% formaldehyde, stained red with phalloidin-tetramethylrhodamine isothiocyanate (phalloidin-TRITC) and examined at a magnification of 100× using CLSM, (Carl Zeiss, Jena, Germany). Samples were excited with 488 nm (green) and 543 nm (red) laser lines, at an acquisition resolution of 1024 × 1024. Pinhole diameters were set to 1 airy unit (AU). Confocal images were edited and quantitatively analysed using ImageJ software (Version 1.48), a public domain image analysis programme, which was developed at the National Institute of Health (NIH) Bethesda, Maryland, USA.

### 4.7. Confocal Microscopy of the Interaction of PLGA-PEG NPs with ADP-Activated PRP

ADP (20 µM) was added to PRP for 5 min. Then, NPs at the three selected sizes (~113, ~321, and ~585 nm) were added for a further 5 min, at room temperature. Samples were, then, fixed with 1% formaldehyde and stained with phalloidin-TRITC. A 200 µL volume of each platelet suspension was layered onto glass slides. Coverslips were mounted using aqueous fluorescence mounting medium DAKO^®^. Imaging of samples using CLSM was performed on a Zeiss LSM 510 confocal microscope (Carl Zeiss, Jena, Germany), fitted with a 100×/1.4 Plan Apochromat (Carl Zeiss, Jena, Germany),. oil-immersion objective. Samples were excited with 488 nm (green) and 543 nm (red) laser lines, at an acquisition resolution of 1024 × 1024. Pinhole diameters were set to 1 airy unit (AU). Confocal images were edited and quantitatively analysed using ImageJ software (Version 1.48), a public domain image analysis programme, which was developed at the National Institute of Health (NIH) Bethesda, MD, USA.

### 4.8. Statistical Analysis

Statistical analysis was carried out using GraphPad Prism (version 7.00 for Windows; GraphPad Software, San Diego, CA, USA). Unpaired Student’s *t*-test and one-way ANOVA followed by post-hoc analysis using Tukey tests were used to determine differences between samples and groups. A *p*-value < 0.05 was considered to be statistically significant.

## 5. Conclusions

The data in this study demonstrate there is a lack of platelet reactivity in PRP towards PLGA-PEG NPs in the absence of an agonist. Upon addition of the agonist, ADP, a “no effect” of NPs of 113 nm on % PA of platelets in PRP was observed. However, in the presence of the larger sized NPs of 321 and 585 nm, at higher NP concentrations, a noticeable decrease in ADP-induced % PA of platelets in PRP was shown. Additionally, the flow cytometry analysis showed no effect of the PLGA-PEG NPs on platelet activation profile, as indicated by the expression of CD62P on both resting and activated platelets. Moreover, no alteration in resting or activated platelet morphology was found upon incubation with NPs. This suggests no influence on platelet reactivity by the NPs irrespective of the duration of incubation or NP size. This lack of platelet reactivity in this study suggests that these PLGA-PEG NPs may be of significant clinical relevance; they possibly have potential as promising vascular-targeted nanocarriers with no risk of alteration of platelets activity, for application in the treatment of various platelet-related disorders or indeed in inflammation-related diseases.

## Figures and Tables

**Table 1:** Effect of size and concentration of Poly-lactide-co-glycolide-polyethylene glycol (PLGA-PEG) nanoparticles (NPs) on percent platelet aggregation (% PA) of platelets in platelet-rich plasma (PRP) following incubation with 20 µM adenosine diphosphate (ADP) for 12 min.

PLGA-PEG NPs (mg/mL)	% PA (113 nm)	% PA (321 nm)	% PA (585 nm)
0 (Control)	82.25 ± 2.75	87.25 ± 2.87	86.20 ± 5.30
0.01	94.75 ± 5.23	94 ± 6.13	95 ± 4.26
0.05	92.50 ± 4.21	89.50 ± 1.93	92.25 ± 3.09
0.1	93.75 ± 7.15	85.50 ± 1.93	89.25 ± 4.87
0.25	101.5 ± 5.90	62.75 ± 3.40 ****	83.40 ± 2.56
0.5	96.50 ± 2.60	36.25 ± 3.28 ****	57.6 ± 2.87 ****
1	88.60 ± 5.48	15.80 ± 2.63 ****	39.75 ± 3.42 ****
1.5	95.67 ± 4.37	6.25 ± 3.09 ****	15.75 ± 2.56 ****
2.2	92.5 ± 4.21	3.6 ± 1.03 ****	9.50 ± 1.44 ****

Data are the average of *n* = 4–5 ± SEM. **** *p* < 0.0001 as compared with 20 µM ADP and PRP (control), one-way ANOVA, and unpaired student *t*-test.

## References

[1] Zhang L., Chan J.M., Gu F.X., Rhee J.W., Wang A.Z., Radovic-Moreno A.F., Alexis F., Langer R., Farokhzad O.C. (2008). Self-assembled lipid-polymer hybrid nanoparticles: A robust drug delivery platform. ACS Nano.

[2] Ramtoola Z., Lyons P., Keohane K., Kerrigan S.W., Kirby B.P., Kelly J.G. (2011). Investigation of the interaction of biodegradable micro- and nanoparticulate drug delivery systems with platelets. J. Pharm. Pharmacol..

[3] He C., Hu Y., Yin L., Tang C., Yin C. (2010). Effects of particle size and surface charge on cellular uptake and biodistribution of polymeric nanoparticles. Biomaterials.

[4] Chan J.M., Zhang L., Yuet K.P., Liao G., Rhee J.-W., Langer R., Farokhzad O.C. (2009). PLGA-lecithin-PEG core-shell nanoparticles for controlled drug delivery. Biomaterials.

[5] Moghimi S.M., Hunter A.C., Murray J.C. (2001). Long-Circulating and Target-Specific Nanoparticles: Theory to Practice. Pharmacol. Rev..

[6] Stevens K.N.J., Crespo-Biel O., van den Bosch E.E.M., Dias A.A., Knetsch M.L.W., Aldenhoff Y.B.J., van der Veen F.H., Maessen J.G., Stobberingh E.E., Koole L.H. (2009). The relationship between the antimicrobial effect of catheter coatings containing silver nanoparticles and the coagulation of contacting blood. Biomaterials.

[7] Jun E.A., Lim K.M., Kim K., Bae O.N., Noh J.Y., Chung K.H., Chung J.H. (2011). Silver nanoparticles enhance thrombus formation through increased platelet aggregation and procoagulant activity. Nanotoxicology.

[8] Li X., Radomski A., Corrigan O.I., Tajber L., Menezes F.D.S., Endter S., Medina C., Radomski M.W. (2009). Platelet compatibility of PLGA, chitosan and PLGA-chitosan nanoparticles. Nanomedicine.

[9] Bakhaidar R., Green J., Alfahad K., Samanani S., Moollan N., O’Neill S., Ramtoola Z. (2019). Effect of size and concentration of PLGA-PEG nanoparticles on activation and aggregation of washed human platelets. Pharmaceutics.

[10] Marx R.E. (2001). Platelet-rich plasma (PRP): What is PRP and what is not PRP?. Implant Dent..

[11] Lacci K.M., Dardik A. (2010). Platelet-rich plasma: Support for its use in wound healing. Yale J. Biol. Med..

[12] Jarvis G.E., Humphries R.G., Robertson M.J., Leff P. (2000). ADP can induce aggregation of human platelets via both P2Y1 and P2T receptors. Br. J. Pharmacol..

[13] Hollopeter G., Jantzen H.M., Vincent D., Li G., England L., Ramakrishnan V., Yang R.B., Nurden P., Nurden A., Julius D. (2001). Identification of the platelet ADP receptor targeted by antithrombotic drugs. Nature.

[14] Zhou L., Schmaier A.H. (2005). Platelet Aggregation Testing in Platelet-Rich Plasma Description of Procedures with the Aim to Develop Standards in the Field. Am. J. Clin. Pathol..

[15] Nemmar A., Hoet P.H.M., Vanquickenborne B., Dinsdale D., Thomeer M., Hoylaerts M.F., Vanbilloen H., Mortelmans L., Nemery B. (2002). Passage of inhaled particles into the blood circulation in humans. Circulation.

[16] Gao X., Cui Y., Levenson R.M., Chung L.W.K., Nie S. (2004). In vivo cancer targeting and imaging with semiconductor quantum dots. Nat. Biotechnol..

[17] Samuel S.P., Santos-Martinez M.J., Medina C., Jain N., Radomski M.W., Prina-Mello A., Volkov Y. (2015). CdTe quantum dots induce activation of human platelets: Implications for nanoparticle hemocompatibility. Int. J. Nanomed..

[18] Dobrovolskaia M.A., Aggarwal P., Hall J.B., McNeil S.E. (2008). Preclinical studies to understand nanoparticle interaction with the immune system and its potential effects on nanoparticle biodistribution. Mol. Pharm..

[19] Shrivastava S., Bera T., Singh S.K., Singh G., Ramachandrarao P., Dash D. (2009). Characterization of Antiplatelet Properties of Silver Nanoparticles. ACS Nano.

[20] Jiang W., Kim B.Y.S., Rutka J.T., Chan W.C.W. (2008). Nanoparticle-mediated cellular response is size-dependent. Nat. Nanotechnol..

[21] Miyamoto M., Sasakawa S., Ozawa T., Kawaguchi H., Ohtsuka Y. (1989). Platelet aggregation induced by latex particles. I. Effects of size, surface potential and hydrophobicity of particles. Biomaterials.

[22] Lundqvist M., Stigler J., Elia G., Lynch I., Cedervall T., Dawson K.A. (2008). Nanoparticle size and surface properties determine the protein corona with possible implications for biological impacts. Proc. Natl. Acad. Sci. USA.

[23] Jackson S.P. (2007). The growing complexity of platelet aggregation. Blood.

[24] Corbalan J.J., Medina C., Jacoby A., Malinski T., Radomski M.W. (2012). Amorphous silica nanoparticles aggregate human platelets: Potential implications for vascular homeostasis. Int. J. Nanomed..

[25] Lundqvist M., Augustsson C., Lilja M., Lundkvist K., Dahlbäck B., Linse S., Cedervall T. (2017). The nanoparticle protein corona formed in human blood or human blood fractions. PLoS ONE.

[26] Tenzer S., Docter D., Kuharev J., Musyanovych A., Fetz V., Hecht R., Schlenk F., Fischer D., Kiouptsi K., Reinhardt C. (2013). Rapid formation of plasma protein corona critically affects nanoparticle pathophysiology. Nat. Nanotechnol..

[27] Huang R.B., Mocherla S., Heslinga M.J., Charoenphol P.P., Eniola-Adefeso O. (2010). Dynamic and cellular interactions of nanoparticles in vascular-targeted drug delivery (review). Mol. Membr. Biol..

[28] Mayer A., Vadon M., Rinner B., Novak A., Wintersteiger R., Fröhlich E. (2009). The role of nanoparticle size in hemocompatibility. Toxicology.

[29] White J.G. (2005). Platelets are covercytes, not phagocytes: Uptake of bacteria involves channels of the open canalicular system. Platelets.

[30] Deb S., Patra H.K., Lahiri P., Dasgupta A.K., Chakrabarti K., Chaudhuri U. (2011). Multistability in platelets and their response to gold nanoparticles. Nanomed. Nanotechnol. Biol. Med..

[31] Youssefian T., Drouin A., Massé J.M., Guichard J., Cramer E.M. (2002). Host defense role of platelets: Engulfment of HIV and Staphylococcus aureus occurs in a specific subcellular compartment and is enhanced by platelet activation. Blood.

[32] Gupalo E., Kuk C., Qadura M., Buriachkovskaia L., Othman M. (2013). Platelet-adenovirus vs. inert particles interaction: Effect on aggregation and the role of platelet membrane receptors. Platelets.

